# Filling Gaps in Biodiversity Knowledge for Macrofungi: Contributions and Assessment of an Herbarium Collection DNA Barcode Sequencing Project

**DOI:** 10.1371/journal.pone.0062419

**Published:** 2013-04-30

**Authors:** Todd W. Osmundson, Vincent A. Robert, Conrad L. Schoch, Lydia J. Baker, Amy Smith, Giovanni Robich, Luca Mizzan, Matteo M. Garbelotto

**Affiliations:** 1 Forest Pathology and Mycology Laboratory, Department of Environmental Science, Policy and Management, University of California, Berkeley, California, United States of America; 2 Centraalbureau voor Schimmelcultures, CBS-KNAW Fungal Biodiversity Centre, Utrecht, The Netherlands; 3 National Center for Biotechnology Information, National Library of Medicine, National Institutes of Health, Bethesda, Maryland, United States of America; 4 Venice Museum of Natural History, Venice, Italy; Fordham University, United States of America

## Abstract

Despite recent advances spearheaded by molecular approaches and novel technologies, species description and DNA sequence information are significantly lagging for fungi compared to many other groups of organisms. Large scale sequencing of vouchered herbarium material can aid in closing this gap. Here, we describe an effort to obtain broad ITS sequence coverage of the approximately 6000 macrofungal-species-rich herbarium of the Museum of Natural History in Venice, Italy. Our goals were to investigate issues related to large sequencing projects, develop heuristic methods for assessing the overall performance of such a project, and evaluate the prospects of such efforts to reduce the current gap in fungal biodiversity knowledge. The effort generated 1107 sequences submitted to GenBank, including 416 previously unrepresented taxa and 398 sequences exhibiting a best BLAST match to an unidentified environmental sequence. Specimen age and taxon affected sequencing success, and subsequent work on failed specimens showed that an ITS1 mini-barcode greatly increased sequencing success without greatly reducing the discriminating power of the barcode. Similarity comparisons and nonmetric multidimensional scaling ordinations based on pairwise distance matrices proved to be useful heuristic tools for validating the overall accuracy of specimen identifications, flagging potential misidentifications, and identifying taxa in need of additional species-level revision. Comparison of within- and among-species nucleotide variation showed a strong increase in species discriminating power at 1–2% dissimilarity, and identified potential barcoding issues (same sequence for different species and vice-versa). All sequences are linked to a vouchered specimen, and results from this study have already prompted revisions of species-sequence assignments in several taxa.

## Introduction

With recent estimates of diversity ranging from approximately 720,000 [1] to over 5.1 million [2] species–of which only approximately 99,000 have been described [3], [4]–the status of Fungi as a poorly known group of organisms is well-established and frequently discussed in the scientific literature [3]. An even larger unknown is the ecology and basic biology of fungal species, both described and undescribed: knowledge of their geographic range, host range, diversity of life cycle stages, and community ecology remains fragmentary.

The ubiquity, high diversity and often cryptic manifestations of fungi frequently necessitate the use of molecular tools for detecting and identifying them in the environment. By far, the most widely used molecular marker for this purpose is the nuclear ribosomal RNA internal transcribed spacer (ITS) region [5], [6]. In recent comparisons, ITS has been demonstrated to outperform other tested markers in terms of overall PCR amplification success, sequencing success, and species resolution for many groups of fungi [5], [7], [8]. In addition, a significant amount of analytical infrastructure, including sequence processing and quality-checking tools as well as curated reference databases, is tailored to this locus [9]–[16]. These features, as well as the momentum provided by the large number of ITS sequences currently in public sequence databases, have led to formal recognition of ITS as the official DNA barcoding locus for fungi by the Consortium for the Barcode of Life [5], [8].

Because of the ephemeral nature of their sexual sporulating structures, the macrofungi (“mushrooms”, “cup fungi”, etc.) share with microfungi the problem of cryptic manifestation during much of their life cycle. As a result, an ecological understanding of these organisms has benefited significantly from the application of DNA-based identification to hyphae, mycorrhizal root tips, or other morphologically cryptic structures [17], [18]. Nonetheless, the ability to obtain DNA barcode sequences from morphologically identifiable, vouchered sporocarps is a strong advantage to the study of these taxa. For ecological studies based on environmental DNA sequences, a reference set of sequences obtained from taxonomically verified sporocarps from the same site is ideal for taxonomic identification [19]–21. However, such data are often not available, necessitating reference to data archived in the databases of the International Nucleotide Sequence Database Collaboration (INSDC) or curated data sets in the UNITE database [11]. Although the utility of such comparisons depends upon the level of database coverage, such coverage is poor for fungi. For example, Hibbett and colleagues [22] observed that 74.4% of newly described fungal species catalogued by *Index Fungorum* from 1999 to 2009 were not represented by molecular sequences in GenBank. Similarly, Brock et al. [23] extrapolated, based on sampling of the Royal Botanic Gardens, Kew herbarium, that approximately 70% of the fungal taxa in herbarium collections were not represented in the INSDs. Efforts to reduce the incomplete taxon coverage of the public sequence databases will increase the utility of these tools for ecological and taxonomic inference.

The role of natural history collections as important sources for DNA barcode data has been previously demonstrated [23], [24]. An additional advantage is that herbaria can offer sampling of related, morphologically verified species collected within a close geographic distance, allowing assessment of barcode gaps and other measures of barcode performance for many taxa simultaneously. In the present study, we conducted a large-scale barcoding effort aimed at representing the diversity of macrofungal collections housed in the herbarium of the Venice Museum of Natural History, Italy. The Venice Museum hosts one of the largest and best-preserved fungal collections in Italy with more than 25,000 samples, representing over 6,000 species of fungi. Collections are mostly recent (1980s to present) and, though largely collected within a single country, represent wide habitat diversity from the Alps to the central plains, Apennines, and Mediterranean coast. The herbarium's strong link to Italy's largest amateur mycological society, Associazione Micologica Bresadola, illustrates the potential for amateur-professional collaborations to contribute to biodiversity science, and provide a means for bidirectional exchange of morphological and molecular taxonomic data to enhance the accuracy of names on both the herbarium collections and their corresponding DNA sequences. In addition to generating over 1100 ITS barcode sequences, we conducted analyses to quantify the contribution of the project to filling gaps in GenBank and to assess overall patterns of barcode discrimination. We also developed a heuristic framework for assessing the overall performance of a large-scale barcoding project from the ground up–including specimen classification, identification of factors affecting success of PCR amplification, and the determination of taxonomic groups most in need of species-level revision. Metrics used in this framework included (1) proper assignment of specimens to genera based on sequence similarity searches, distance-based clustering, and ordination; (2) correlation of taxon and specimen age to PCR amplification success; (3) the effect on PCR success and barcode discrimination of ITS1 or ITS2 mini barcodes; (4) potential identification errors or taxonomic questions based on the presence/absence of barcode gaps between taxa and on the prevalence of false negative and false positive barcode identification errors.

## Materials and Methods

### Specimen Collection

Sample collection focused on taxonomic breadth in an attempt to obtain barcode sequences for the largest possible proportion of the approximately 6000 species in the collection, while including some limited within-taxon replication in order to assess within- vs. among-species nucleotide variation (“barcode gaps”). Species coverage predominantly consisted of Agaricales (Basidiomycota) with additional coverage within other Basidiomycota orders, as well as Ascomycota and Glomeromycota (Table S1). Dried herbarium samples ranging in age from the 1980s to 2005 were sampled by removing a small piece (8–64 cubic mm) of sporocarp tissue using a sterilized forceps, attempting to avoid commonly contaminated sites (e.g., the upper surface of the mushroom cap) whenever possible. No permits were required for this study. Permission of the herbarium was obtained for destructive sampling of specimens to allow for DNA extraction, and this study did not involve endangered or protected species.

### DNA Extraction, PCR and Sequencing

DNA was extracted from specimens following Ivors et al. [25]. Briefly, dried sporocarp samples were pulverized using a bead mill, suspended in a CTAB extraction buffer, and subjected to 3 rounds of freeze-thaw consisting of alternating 3 min treatments in dry ice and a 70°C heating block, followed by a 30 min incubation at 70°C. Samples were subsequently treated with phenol: chloroform: isoamyl alcohol (25∶24∶1) and centrifuged for 15 min at 13,000× g, then DNA was purified from the supernatant using the GeneClean Turbo kit (QBiogene, Inc.).

PCR reactions were conducted using the primers ITS1F [26] and ITS4 [27], amplifying the ITS1+5.8S+ITS2 portion of the nuclear ribosomal DNA repeat region. A subset of samples not yielding ITS1F/ITS4 amplicons were amplified using the primer pairs ITS1F and ITS2 [27], which amplifies only the ITS1 region. PCR reaction mixtures were prepared in 25 µl volumes including 5 µl 5X PCR buffer (GoTaq Flexi; Promega Inc., Madison, WI, USA), 2.5 µl dNTPs (2 mM/L), 2.5 µl BSA (2.5 mg/mL), 2 µl MgCl2 (25 mM/L), 1 µl each primer (10 µM/L), 0.2 µl GoTaq Flexi DNA polymerase (5 U/ µl), 20 ng template DNA, and sterile ddH2O to reach 25 µl total. Cycling parameters were as follows: 94°C for 2 min; 30 cycles of 94°C for 30 sec, 55°C for 1 min, and 72°C for 1 min; 72°C for 5 min. PCR products were cleaned using ExoSAP-IT (Affymetrix Inc., Santa Clara, CA). Sequencing reactions were conducted using BigDye 3.1 dye terminator chemistry with the primer pair ITS1F/ITS4 or ITS1F/ITS2, cleaned using ethanol precipitation, and analyzed using an ABI 3130 Genetic Analyzer (Applied Biosystems, Foster City, CA). Sequence contig assembly and editing used Sequencher 4.7 (Gene Codes Corp., Ann Arbor, MI, USA).

### Data Quality Assessment and Sequence Submission

Given the prevalence of misidentifications in herbarium and culture collections [28], several safeguards against misidentified material were taken. Sampling was performed from a well-curated collection in which many taxonomic groups are maintained by knowledgeable authorities. NCBI BLASTn queries were submitted for each sequence prior to further analyses, and sequences were discarded for which a high-similarity top hit did not belong to the same genus, or to a genus closely related to, the one assigned to the herbarium specimen. Even with these safeguards, however, incorrectly identified sequences could be incorporated in a specimen sequencing effort of this size and breadth. Therefore, proper assignments to families and genera were qualitatively assessed using UPGMA dendrograms and nonmetric multidimensional scaling (NMDS) ordinations based on a matrix of pairwise sequence distances calculated across the entire dataset. These analyses were conducted using the BioloMICS software package (www.bio-aware.com). Based on these results, potentially misidentified specimens were flagged for manual examination and correction.

GenBank sequence submissions were prepared using a custom Perl script for constructing feature annotation tables and the NCBI tbl2asn utility for automating generation of GenBank submission flat files. Specimen data and accession numbers are provided in Table S1.

### Assessment of Factors Affecting PCR Amplification Success

Associations of age and taxon with PCR amplification success rate were assessed using chi-squared tests of independence implemented in JMP v9 (SAS Institute, Cary NC, USA). It was assumed that no significant, systematic differences in storage conditions or preservation methodology existed across the collection. Post hoc assessments were conducted by calculating standardized and adjusted residuals [29], [30] for each cell in the contingency table and comparing these values to a standard normal distribution, applying a Bonferroni correction for multiple row-wise contrasts [31].

Because DNA degradation may be the cause of PCR amplification failure, the potential for shorter “mini barcodes” to increase PCR amplification success without decreasing barcode discrimination was tested as follows. The ITS1 spacer region was amplified using primers ITS1F+ITS2 for 30 randomly selected samples previously negative for full-length ITS1F+ITS4 PCR amplifications from each of the three large and well represented genera *Cortinarius, Russula* and *Mycena*. Mini-barcode discrimination potential was examined by extracting separate ITS1 and ITS2 sequences for each of the 1107 full-length ITS1+5.8S+ITS2 sequences, constructing pairwise distance matrices, and determining Pearson correlations between the ITS1, ITS2, and full-length sequence distance matrices using BioloMICS software.

### Barcode Sequence Analyses

Several analyses were conducted to discern general trends in barcode performance across the dataset and to identify potential identification errors and highlight taxa that warrant further taxonomic study. “Barcode gaps,” i.e., differences in the degree of sequence similarity within and among species, were assessed by calculating pairwise nucleotide differences across the dataset and categorizing each comparison as either intraspecific or interspecific based on the specimen identification. Calculations were retained only for sequence pairs differing by≤70 bp, or 10% of the typical ∼700 bp length of an ITS amplicon, a level determined empirically to yield accurate pairwise sequence alignments. Comparisons between taxonomic and molecular identifications were used to quantify the occurrence of two types of potential barcode classification error: false negative (identical ITS sequences for different morphological species), and false positive (>1 ITS sequence between collections of a single morphological species).

## Results

### Barcode Sequence Generation and Quality Assessment

**Figure 1:**
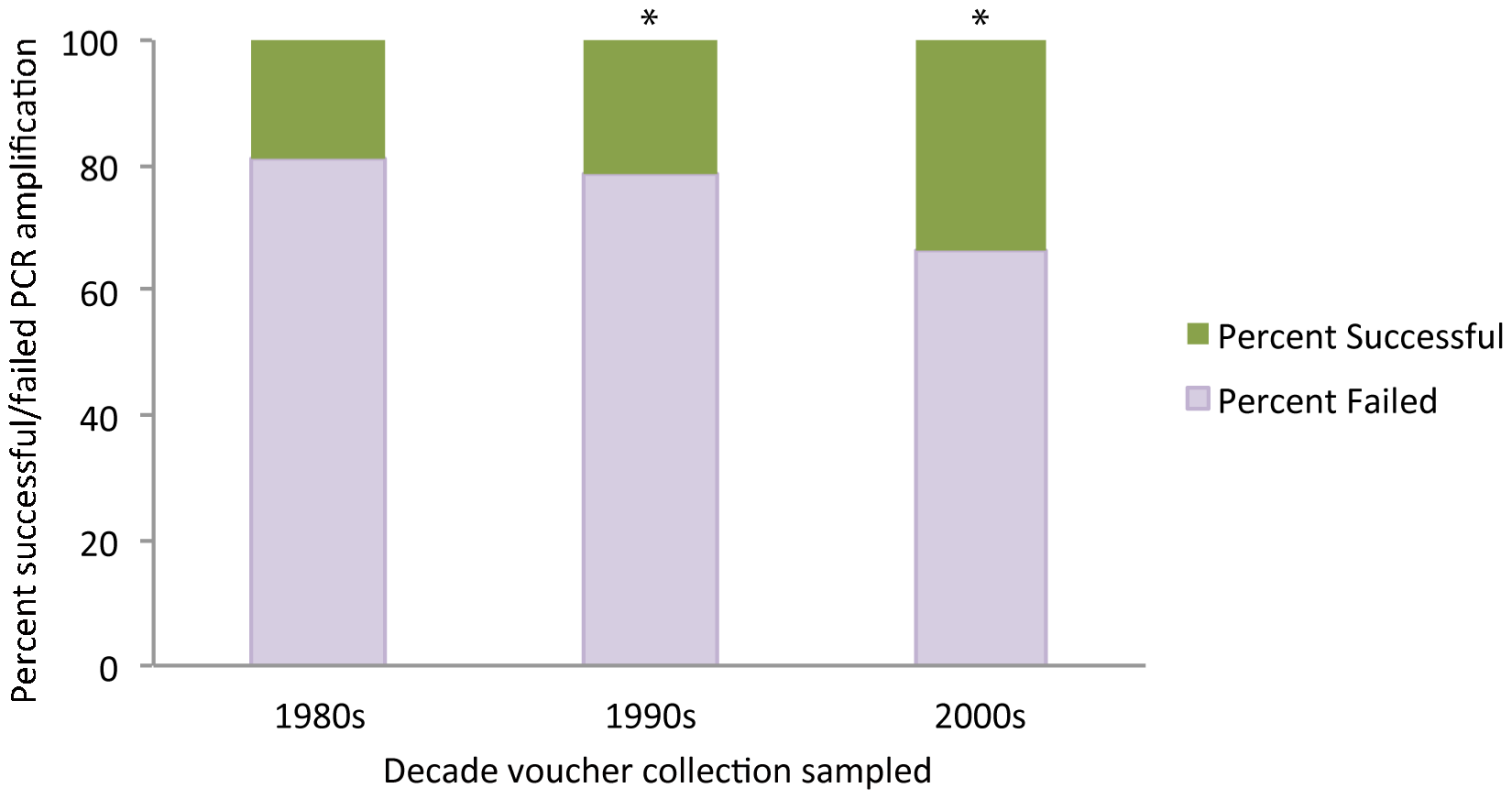
Association between specimen age and PCR amplification success using primers ITS1F and ITS4. Pearson Chi-square test of independence (N = 2763, d.f. = 2) significant at p<0.0001. Asterisks denote categories with standardized adjusted residuals significant at a Bonferroni-corrected α = 0.00833. See Table S2 for data and post hoc test results.

**Figure 2:**
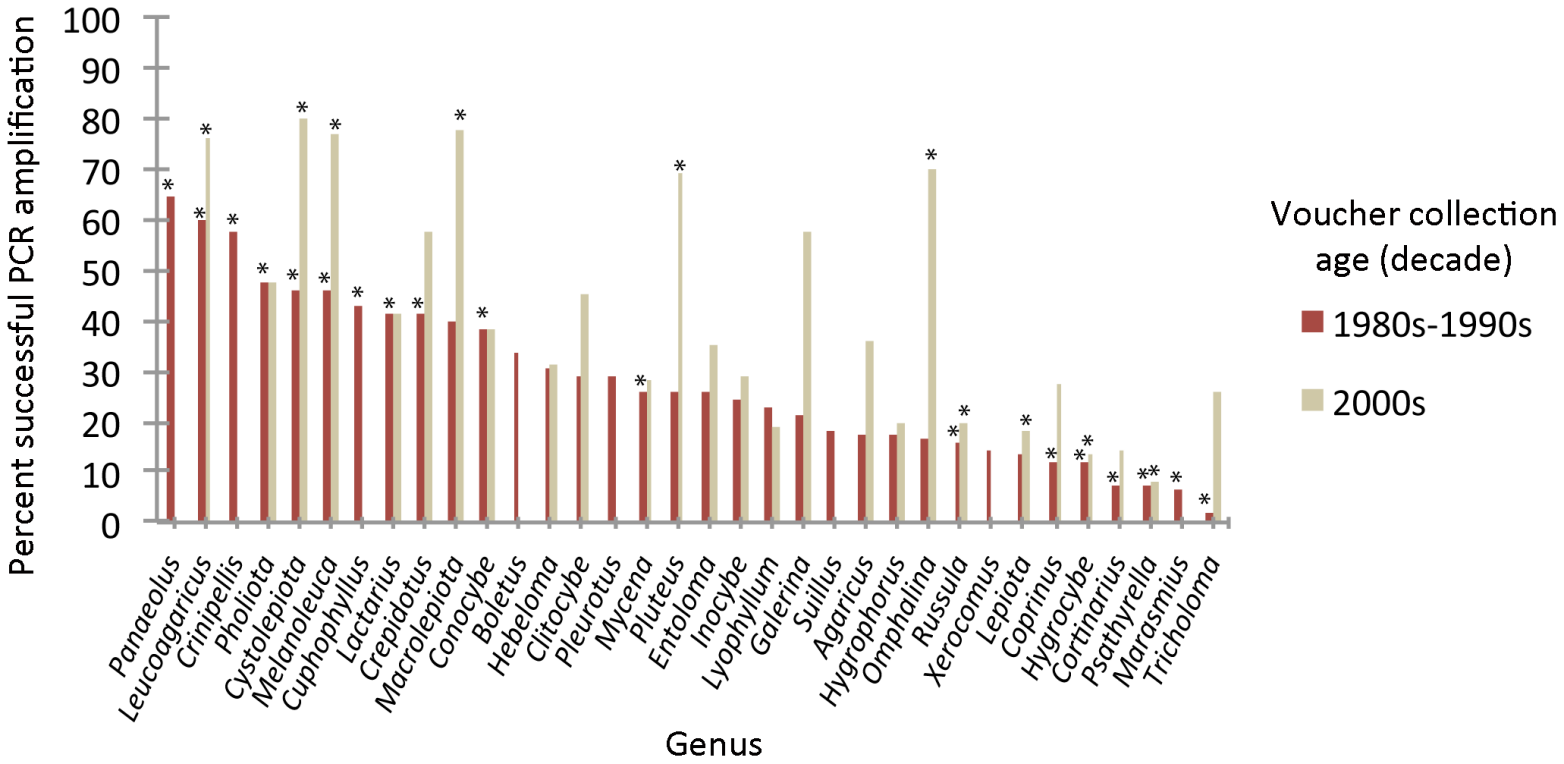
Association between taxon (controlled for specimen age) and PCR amplification success using primers ITS1F and ITS4. Pearson Chi-square test of independence: p<0.0001. Asterisks denote categories with standardized adjusted residuals significant α = 0.05. See Table S3 for data and post hoc test results.

**Figure 3:**
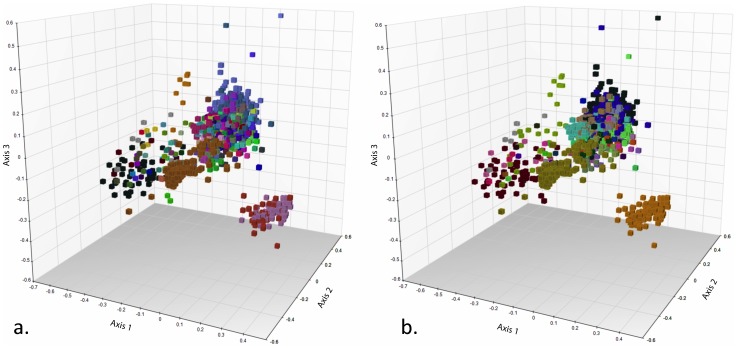
Assessment of concordance between taxonomic (herbarium determination) and DNA similarity assignment using nonmetric multidimensional scaling (NMDS) ordination of the pairwise genetic distance matrix. a. Data symbols coded by genus. b. Data symbols coded by family.

Of approximately 5000 specimens sampled, 2763 were PCR positive using primers ITS1F and ITS4. Specimen age was significantly correlated with PCR success, with older specimens exhibiting lower levels of successful amplification (Figure 1; Table S2). After correction for specimen age, taxonomic identity at the genus level still exhibited a strong correlation with PCR amplification success (Figure 2; Table S3). Of the 2763 PCR positive specimens, approximately 1600 yielded high quality sequences in one or both directions. Following initial quality checking using NCBI BLASTn searches, 1107 bidirectional sequences representing 936 unique taxon names were submitted to GenBank (Table S1). Clustering of sequences with congeneric and confamilial accessions in UPGMA dendrograms (Figures S1 and S2) and NMDS ordinations (Figure 3) indicated overall high quality of specimen identifications, while identifying accessions requiring additional scrutiny. Causes for problematic accessions appeared to include (1) misidentifications or mixed samples; e.g., inclusion of a *Helvella ephippium* accession within *Cortinarius*; (2) nomenclatural issues; e.g., several *Coprinus* specimens lacking updated genus and family classifications; and (3) instances where either polyphyly or the distance metric produced multiple UPGMA clusters; e.g., multiple clusters containing *Entoloma* species (Figures S1, S2).

Of the 936 unique taxon names in the dataset, 416 (44.4%) were previously unrepresented by ITS sequences in GenBank. Of the 1107 sequences, 398 (36%) exhibited a best BLAST match to an unidentified environmental sequence in GenBank.

### Generation and Performance of Mini-barcodes

**Figure 4:**
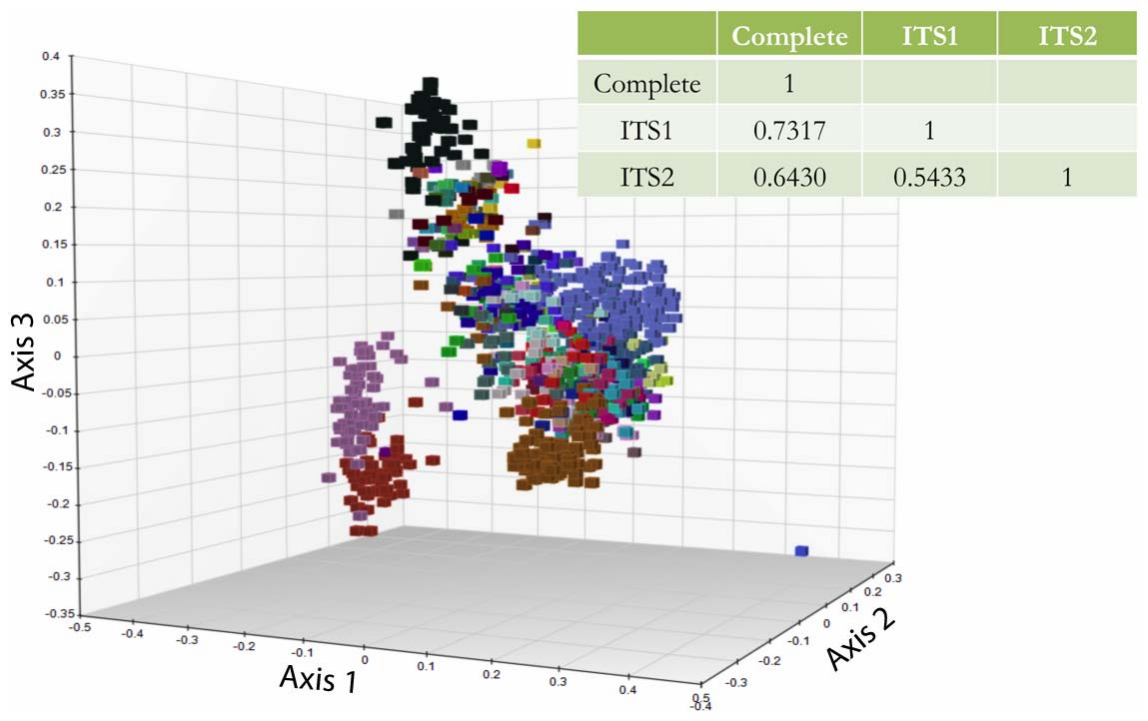
Classification potential of ITS mini-barcodes. NMDS ordination of genetic distances based on ITS1 sequences, with symbols colored by genus. Table inset shows Pearson correlation coefficients between pairwise genetic distance matrices generated for the two spacer regions (ITS1 and ITS2) separately and the full-length sequences from the 1107-sequence dataset.

Distance matrix correlation of each of the 2 spacer regions compared to the full-length sequences for the 1107 sequence dataset indicated a higher correlation for ITS1 than ITS2 to the full-length sequences (Figure 4). NMDS ordination qualitatively indicated that ITS1 mini-barcode sequences properly placed specimens into genera (Figure 4). Using primers that amplify the ITS1 spacer for accessions that failed for full length ITS1+5.8S+ITS2 amplification, PCR amplification success was increased to 90–100%, and sequencing success was increased slightly (13% in *Mycena*) to significantly (80% in *Cortinarius*; 90% in *Russula*; Table 1).

**Table 1:** Improvement of PCR and sequencing success rates using ITS1 mini-barcodes.

Genus	PCR Positive	Sequence Positive
*Cortinarius*	30/30 (100%)	24/30 (80%)
*Russula*	30/30 (100%)	27/30 (90%)
*Mycena*	27/30 (90%)	4/30 (13%)

PCR and sequencing success rates are shown for 30 randomly-selected samples from 3 macrofungal genera; samples were previously negative for full-length ITS1+5.8S+ITS2 amplification.

### Barcode Discrimination Analyses

**Figure 5:**
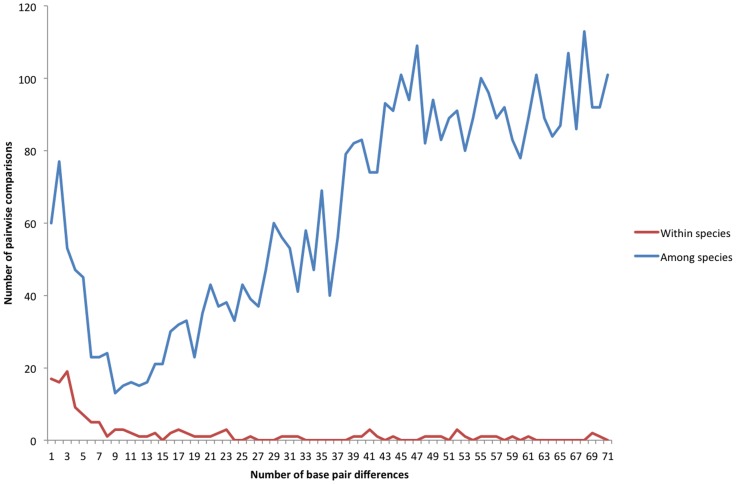
Plot of within- and between-species nucleotide divergence (bp).

Within-species nucleotide divergence exhibited a sharp drop between 6 and 14 bp, corresponding to approximately 1–2% of the common ITS amplicon length of approximately 700 bp (Figure 5). For all levels of nucleotide divergence greater than 0 bp, most instances occurred between, rather than within, morphological species. However, although the presence of a universal barcode gap–indicated by a hiatus between within-species and among-species curves–is suggested by a strongly upward-trending interspecific curve and strongly downward-trending intraspecific curve, a definitive hiatus is lacking due to a significant number of among-species comparisons that exhibit little or no nucleotide divergence (Figure 5). False negative designations occurred for 60 species pairs. Of these, 59 pairs represented congeneric species, thus likely representing synonyms, species complexes or minor misidentifications. The other pair consisted of 1 epigeous- sequestrate confamilial pair (*Leucoagaricus medioflavoides* + *Endoptychum agaricoides*). For instances of pairs exhibiting 1 bp difference but not belonging to the same taxonomic species (n = 77), 74 represented congeners, one a ‘moderate’ misidentification (*Pholiotina* vs. *Galerina*), and two ‘major’ misidentifications or mixed samples (*Boletus* vs. *Inocybe*; *Sarcosphaera* vs. *Psathyrella*).

## Discussion

DNA sequencing from environmental samples has brought about a major shift in the composition of fungal sequences in public DNA sequence databases from overwhelmingly specimen-based to increasingly dominated by environmental sequences [22]. The lack of taxonomic overlap between these two types of sequences in public databases diminishes the potential impact of molecular ecological studies by creating a disconnect between contemporary studies and the wealth of mycological knowledge obtained through over two centuries of classical research in taxonomy, ecology, and pathology. Perhaps nowhere is this disconnect as evident–and as avoidable–as in the macrofungi, given their conspicuous morphological structures. A large number of described macrofungal species remain unrepresented by DNA sequences in public databases [22], [23]. In the present study, we engaged in a large-scale herbarium DNA barcoding project with the goal of increasing this database representation while simultaneously investigating potential issues and assessing the performance of such a project. Our heuristic framework for assessing the overall performance of the project covered aspects from specimen identification to determining overall trends in the data to identifying those taxa that are most in need of species-level revision by taxonomic specialists, rapidly and at the whole-dataset level. Our analyses represent a diverse selection (976 unique taxa) of macrofungi, particularly among the Agaricales.

Specimen identification is a significant concern in any broad-scale museum sequencing project. In addition to standard precautions regarding specimen identification (in our case, drawing upon a relatively small, high quality, curated collection) and quality checking, we qualitatively assessed the general quality of the dataset using dataset-wide distance-based clustering methods targeted at the genus and family levels. Applying these methods to the species level would require the inclusion of taxonomic benchmark sequences in an analysis. Two possible sources for such benchmarks include sequences deposited in GenBank or sequences of Type collections. Given known issues of sequence misidentification in public sequence databases [28], the former source is suboptimal except for initial quality checking. The Type collection approach is untenable in many cases, both in terms of additional sequencing effort and in terms of the problem of missing or old collections. While distance-based metrics do not provide a solid basis for barcode identification [32]–[35], they can provide a heuristic that is rapid, very often corresponds well to existing classifications, and does not require a multiple sequence alignment across all specimens in a large and heterogeneous dataset. We therefore advocate the use of these methods for rapid estimation of data quality, but not for species identification or diagnosis. Ultimately, DNA barcoding relies upon well-identified, vouchered collections; the analyses that we present have the potential to streamline the process of determining priorities for intensive study of particular taxonomic groups by trained experts. Large-scale herbarium sequencing projects have enormous potential to facilitate taxonomic research as long as adequate precautions are taken to ensure the accuracy of identifications to at least the generic or species-group levels. As an important component of the Venice herbarium project, we have invited a number of taxonomic specialists to access our data beginning immediately after sequence generation. The sequencing of vouchered herbarium collections ensures that data are verifiable and source material is available for further study where warranted.

Low sequencing success rates are common features of herbarium sampling projects [23]. A number of factors may influence PCR amplification success, including specimen age, mode of specimen preservation, and biological characteristics of the organism such as spore wall thickness and the presence and types of pigments, polysaccharides, and other biochemicals. We selected taxonomic designation as a useful, albeit imperfect, proxy for these biological characteristics as it (1) provides a single criterion rather than a suite of characteristics to be checked; (2) incorporates these biological characteristics when they are congruent with phylogeny; and (3) is the heuristic most likely to be used by other practitioners when conducting large-scale barcoding studies. In the present study, we demonstrated both age and taxon effects on success of PCR amplification. Sequencing failure from positive PCR amplicons was a comparatively greater source of data loss, with a 40% rate of sequencing success compared to approximately 55% PCR success. A number of factors can contribute to sequencing failure, including sample contamination and sequence divergence between tandem rDNA repeats within a single individual. In cases of initial PCR failure, we demonstrated that rates of PCR and subsequent sequencing success may be substantially improved through the use of shorter amplicons, or mini-barcodes. Although not true for all fungal groups [6], for the large number of predominantly macofungal samples tested here, ITS1 was significantly superior to ITS2 in terms of species discrimination. The ITS1 region carries the added advantage of having a priming site for the fungal-specific primer ITS1F.

Examining within- and among-species nucleotide variation proved valuable both in assessing overall barcode discrimination patterns and as a means of identifying collections or taxonomic groups in need of further scrutiny or taxonomic revision. The overlap in nucleotide similarity levels observed between within-species and among-species classes indicates that a “one size fits all” sequence similarity cutoff for species delimitation–as is often used in molecular ecology studies–may introduce significant error in species diversity estimates. Comparing molecular and morphospecies designations, we detected both false negative (>1 morphological species per ITS sequence) and false positive (>1 ITS sequence per morphological species) species-level assignments. We found that a 1–2% divergence cutoff eliminates most false positives; however, false negatives occur even at 0–1 nucleotide differences. Several explanations exist for these assignments, including minor misidentifications, incompletely resolved taxonomy, cryptic speciation, or incomplete lineage sorting. The generation of aggregate measures across a large dataset can pinpoint problem specimens or taxa in need of further scrutiny to determine whether instances of sequence identity are real or artifacts.

Sequences obtained in this project added 416 species that were previously unrepresented in GenBank and 398 sequences with best BLAST matches to environmental samples. These results highlight the benefit of herbarium sequencing studies for increasing taxonomic coverage in sequence databases and improving the accuracy of taxonomic determination for environmental samples. Given concerns over the decreasing ranks of professional taxonomic mycologists [36], the strong association that the Venice Museum maintains with well-respected taxonomists in the amateur mycological community provides a model for accelerating the pace of taxonomic discovery and translating this taxonomic knowledge to ecology through collaborative DNA barcoding projects.

Large-scale barcoding studies not only benefit from herbarium collections, but in turn provide value to herbaria by increasing their range of relevance between scientific disciplines and by augmenting the ways in which collections can be selected for additional study. For example, potential users could select specific collections for study based on their DNA sequences and/or clade membership rather than on their taxonomic names. Misidentification and out-of-date nomenclature for vouchered collections are problems that can be passed on to their associated sequence accessions. In spite of this issue, however, DNA barcode sequences derived from vouchered material should remain the gold standard for identification of environmental DNA sequences, because re-examining the voucher collections can straightforwardly assess potential misidentification errors. Assuring the accuracy of taxonomic names in both herbarium collections and sequence databases can–and should–involve engaging in a dynamic, bidirectional exchange of taxonomic information between collectors of the two sources of data. Collaboration with taxonomic experts has already improved identifications of a number of the sequences generated during this project.

Increasing the population of vouchered material in public sequence databases will benefit ecology, pathology, and general mycology by fostering links between new ecological insights and the body of knowledge pertaining to previously described species. High throughput environmental sequencing projects will no doubt continue to increase the number of insufficiently identified sequences in public databases. While many of these sequences may represent new species or even higher-order lineages, described taxa are the touchstones necessary for placing this vast, unknown diversity in the context of our existing phylogenetic and intellectual frameworks.

### Additional rescources

An interactive map showing the geographic location of all sequenced accessions, linked to collection data and searchable by taxon, is available for viewing or download from the project website, http://nature.berkeley.edu/garbelotto/english/venice.php. DNA sequence data organized by genera are available for download (in FASTA format) on the project website. We invite researchers to use these sequences in their analyses and provide feedback that would be useful for refining the taxonomic identifications attached to these data; communications should be directed to the corresponding author.

## Supporting Information

Figure S1 **UPGMA dendrogram showing clustering of 1107 ITS sequences obtained from vouchered macrofungal collections in the herbarium of the Venice natural history museum.** Columns to the right of the taxon names indicate clustering by genus and family, facilitating identification of misidentified specimens or taxonomic issues.
(JPG)

Figure S2 **UPGMA dendrogram showing clustering of ITS sequences, containing only those species for which multiple accessions were sequenced.** Non-identical sequences for multiple accessions of a species indicate possible instances of misidentification, intraspecific polymorphism, or cryptic species. Columns to the right of the taxon names indicate clustering by genus and family, facilitating identification of misidentified specimens or taxonomic issues.
(PNG)

Table S1 **Collection data.**
(XLSX)

Table S2 **Post hoc test of results of Χ2 test of independence for PCR success rate by decade. Method for calculation of standardized and adjusted residuals (STARs) is cited in the main article.** Significance of cell-wise residual values was assessed by comparison to a standard normal distribution using a Bonferroni-corrected p-value of 0.05/6 row-wise contrasts = 0.008. Relative contribution was calculated as the proportion of each cell-wise Χ^2^ to the omnibus Χ^2^ statistic.
(DOCX)

Table S3 **Count data for PCR amplification success (Positive) or failure (Negative), and post hoc test of results of Χ2 test of independence, for PCR success rate by taxon (genus) subdivided by decade of specimen collection (1980s–1990s vs. 2000s).** Method for calculation of standardized and adjusted residuals (STARs) is cited in the main article. Significant cell-wise residual values are denoted by * for α = 0.05 and § for Bonferroni-corrected α (0.05/595 row-wise contrasts = 0.000084 for 1980s–1990s collections; 0.05/351 row-wise contrasts = 0.00014 for 2000s collections).
(DOCX)
