# Supplementary figures and images for: Filling Gaps in Biodiversity Knowledge for Macrofungi: Contributions and Assessment of an Herbarium Collection DNA Barcode Sequencing Project
Source: PLoS One. 2013 Apr 30;8(4):e62419. doi: 10.1371/journal.pone.0062419 (PMC3640088; doi:10.1371/journal.pone.0062419)

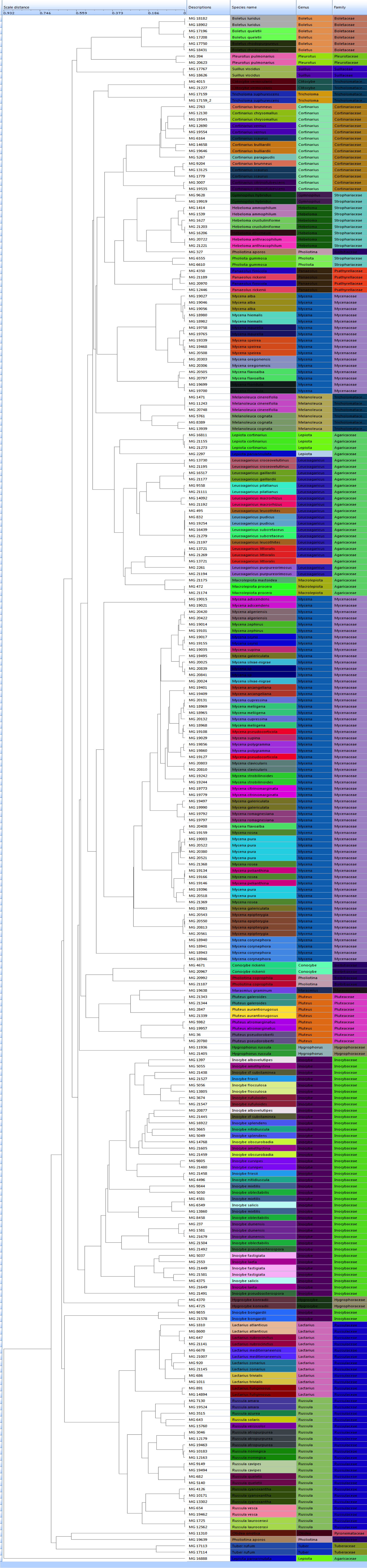

Supplement: Figure S2 — UPGMA dendrogram showing clustering of ITS sequences, containing only those species for which multiple accessions were sequenced. Non-identical sequences for multiple accessions of a species indicate possible instances of misidentification, intraspecific polymorphism, or cryptic species. Columns to the right of the taxon names indicate clustering by genus and family, facilitating identification of misidentified specimens or taxonomic issues. (PNG) [file pone.0062419.s002.png]
