# Supplementary material for: Filling Gaps in Biodiversity Knowledge for Macrofungi: Contributions and Assessment of an Herbarium Collection DNA Barcode Sequencing Project
Source: PLoS One. 2013 Apr 30;8(4):e62419. doi: 10.1371/journal.pone.0062419 (PMC3640088; doi:10.1371/journal.pone.0062419)
Supplement: Table S2 — Post hoc test of results of Χ2 test of independence for PCR success rate by decade. Method for calculation of standardized and adjusted residuals (STARs) is cited in the main article. Significance of cell-wise residual values was assessed by comparison to a standard normal distribution using a Bonferroni-corrected p-value of 0.05/6 row-wise contrasts = 0.008. Relative contribution was calculated as the proportion of each cell-wise Χ2 to the omnibus Χ2 statistic. (DOCX) [file pone.0062419.s004.docx]

| **Decade - Result** | **χ^2^** | **STAR** | **Relative Contribution** |
| --- | --- | --- | --- |
| 1980s – Negative | 0.5026 | 1.45 | 0.011 |
| 1990s – Negative | 2.6778 | 5.74* | 0.061 |
| 2000s – Negative | 7.7572 | -6.58* | 0.178 |
| 1980s – Positive | 1.5048 | -1.45 | 0.034 |
| 1990s – Positive | 8.0171 | -5.74* | 0.184 |
| 2000s – Positive | 23.2247 | 6.58* | 0.532 |
