# Supplementary material for: Filling Gaps in Biodiversity Knowledge for Macrofungi: Contributions and Assessment of an Herbarium Collection DNA Barcode Sequencing Project
Source: PLoS One. 2013 Apr 30;8(4):e62419. doi: 10.1371/journal.pone.0062419 (PMC3640088; doi:10.1371/journal.pone.0062419)
Supplement: Supplementary file 4 [file pone.0062419.s005.docx]

	1980s-1990s				2000s			
**Genus**	**Negative**	**STAR**	**Positive**	**STAR**	**Negative**	**STAR**	**Positive**	**STAR**
Agaricus	29	0.621	6	-0.621	18	-0.246	10	0.246
Amanita	43	3.462*	0	-3.462*	20	3.222*	0	-3.222*
Boletus	10	-1.130	5	1.130	N/A	N/A	N/A	N/A
Clitocybe	30	-1.144	12	1.144	16	-1.311	13	1.311
Conocybe	16	-2.134*	10	2.134*	8	-0.378	5	0.378
Coprinus	52	1.816*	7	-1.816*	8	0.445	3	-0.445
Cortinarius	268	6.238[§]	22	-6.238[§]	12	1.542	2	-1.542
Crepidotus	10	-1.996*	7	1.996*	3	-1.328	4	1.328
Crinipellis	3	-2.309*	4	2.309*	N/A	N/A	N/A	N/A
Cuphophyllus	8	-1.964*	6	1.964*	N/A	N/A	N/A	N/A
Cystolepiota	7	-2.183*	6	2.183*	2	-3.131*	8	3.131*
Entoloma	41	-0.742	14	0.742	24	-0.208	13	0.208
Galerina	11	-0.002	3	0.002	3	-1.328	4	1.328
Hebeloma	23	-1.257	10	1.257	20	0.294	9	-0.294
Hygrocybe	45	1.702*	6	-1.702*	13	1.676*	2	-1.676*
Hygrophorus	29	0.621	6	-0.621	16	1.302	4	-1.302
Inocybe	130	-0.858	41	0.858	78	1.076	32	-1.076
Lactarius	57	-4.888[§]	40	4.888[§]	23	-1.015	16	1.015
Lepiota	46	1.477	7	-1.477	27	*1.914*	6	-1.914*
Leucoagaricus	8	-4.230[§]	12	4.230[§]	5	-4.197[§]	16	4.197[§]
Lyophyllum	10	-0.147	3	0.147	13	1.268	3	-1.268
Macrolepiota	6	-1.437	4	1.437	2	-2.827*	7	2.827*
Marasmius	27	1.920*	2	-1.920*	N/A	N/A	N/A	N/A
Melanoleuca	14	-3.097*	12	3.097*	4	-3.791[§]	13	3.791[§]
Mycena	265	-2.341*	93	2.341*	38	0.841	15	-0.841
Omphalina	10	0.402	2	-0.402	3	-2.457*	7	2.457*
Panaeolus	6	-4.372[§]	11	4.372[§]	N/A	N/A	N/A	N/A
Pholiota	11	-2.944*	10	2.944*	10	-1.291	9	1.291
Pleurotus	5	-0.463	2	0.463	N/A	N/A	N/A	N/A
Pluteus	20	-0.576	7	0.576	9	-4.120[§]	20	4.120[§]
Psathyrella	37	2.168*	3	-2.168*	24	2.844*	2	-2.844*
Russula	118	1.708*	22	-1.708*	66	2.857*	16	-2.857*
Suillus	18	0.371	4	-0.371	N/A	N/A	N/A	N/A
Tricholoma	46	3.264*	1	-3.264*	26	1.007	9	-1.007
Xerocomus	24	0.926	4	-0.926	N/A	N/A	N/A	N/A
